# Supplementary material for: Comprehensive Analysis of CDK1-Associated ceRNA Network Revealing the Key Pathways LINC00460/LINC00525-Hsa-Mir-338-FAM111/ZWINT as Prognostic Biomarkers in Lung Adenocarcinoma Combined with Experiments
Source: Cells. 2022 Apr 4;11(7):1220. doi: 10.3390/cells11071220 (PMC8997540; doi:10.3390/cells11071220)
Supplement: Supplementary file 1 [file cells-11-01220-s001.zip › Supplementary material.pdf]

Supplementary Figure

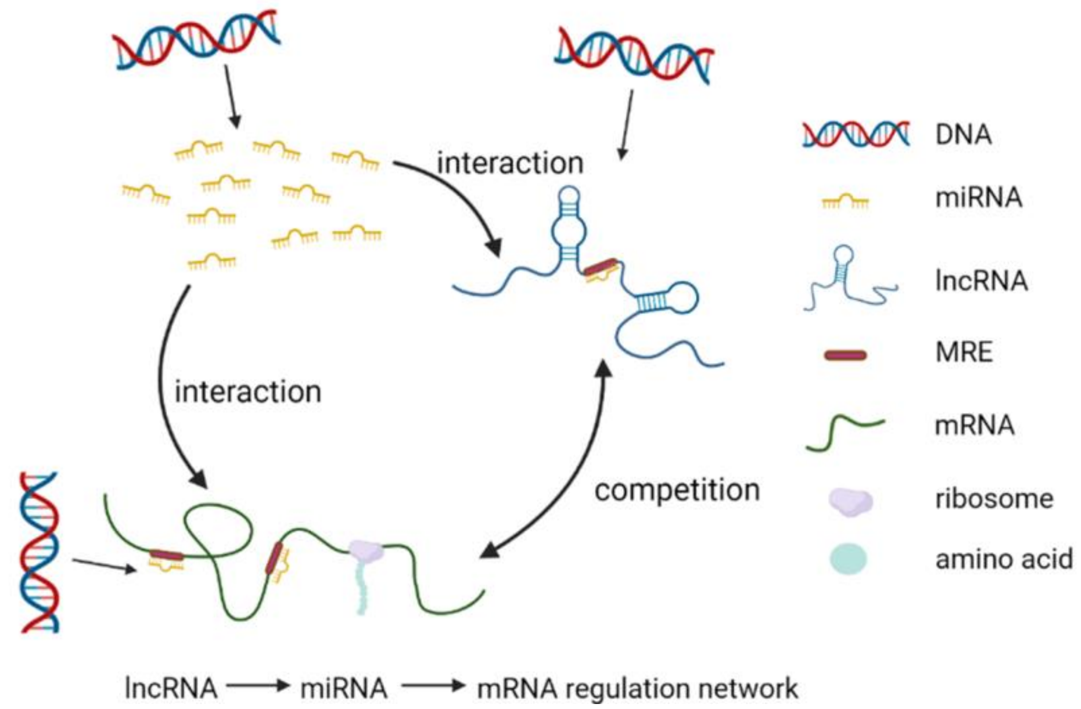

Figure S1. CeRNA regulatory network

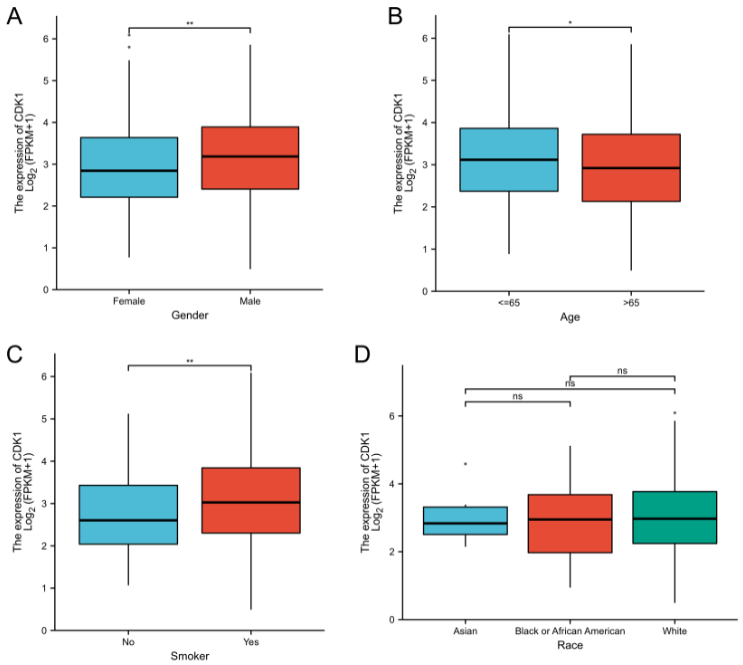

Figure S2. Clinical correlation between CDK1 and LUAD patients. Significance markers: ns, $p \geq 0.05$; *, $p < 0.05$; **, $p < 0.01$; ***, $p < 0.001$. (A-D) The correlation between CDK1 and (A) gender, (B) age, (C) smoking and (D) race.

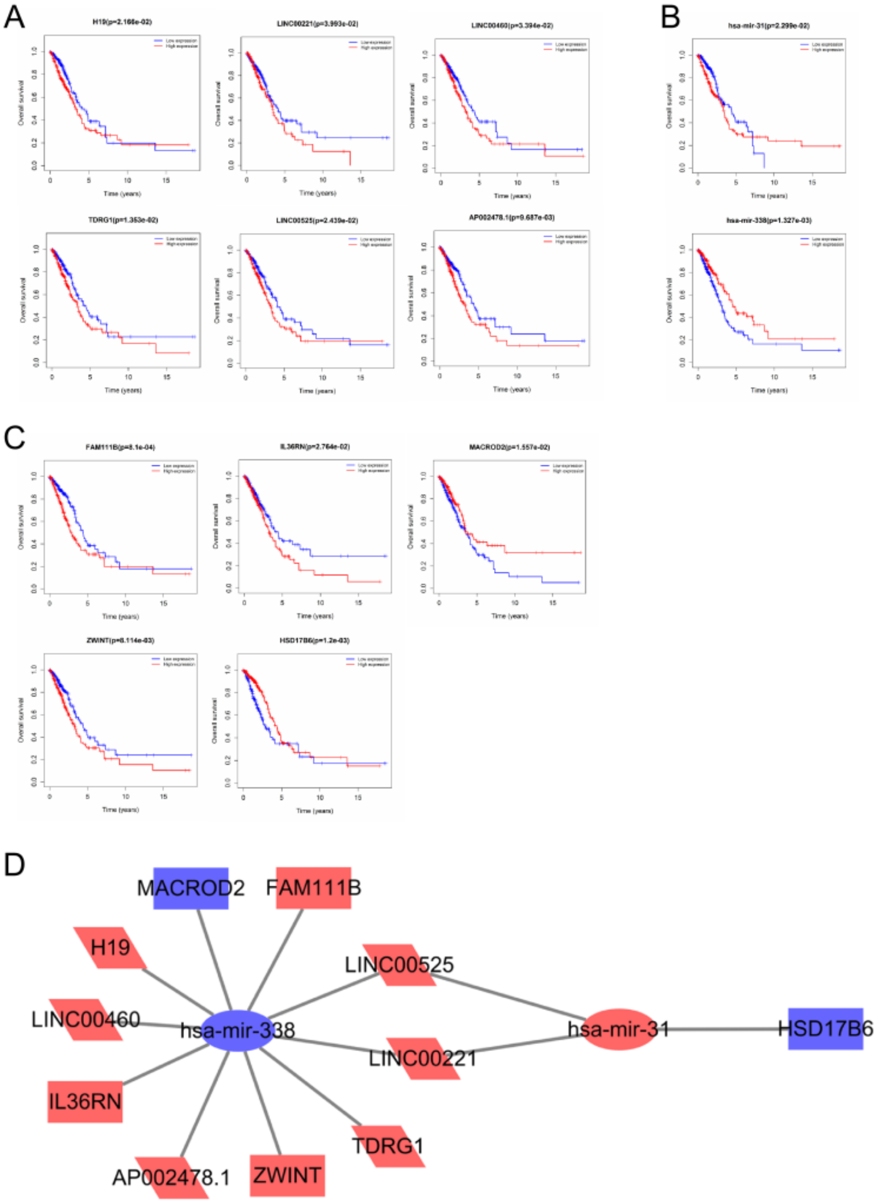

Figure S3. Survival-associated ceRNA networks. Red means up-regulation, Blue means down-regulation. (A-C) Survival-associated DElncRNA, (B) DEmiRNA and (C) DEmRNA. (D) Survival-associated ceRNA network.

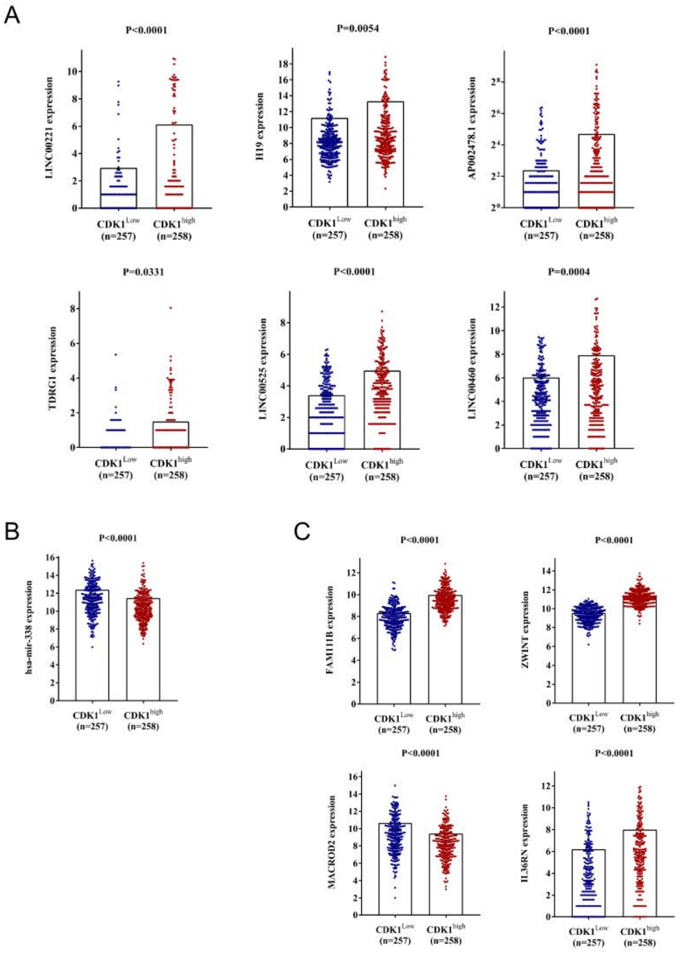

Figure S4. Expression level of DERNAs in CDK1low expression group and CDK1high expression group. (A) DElncRNA. (B) DEmiRNA. (C) DEmRNA.

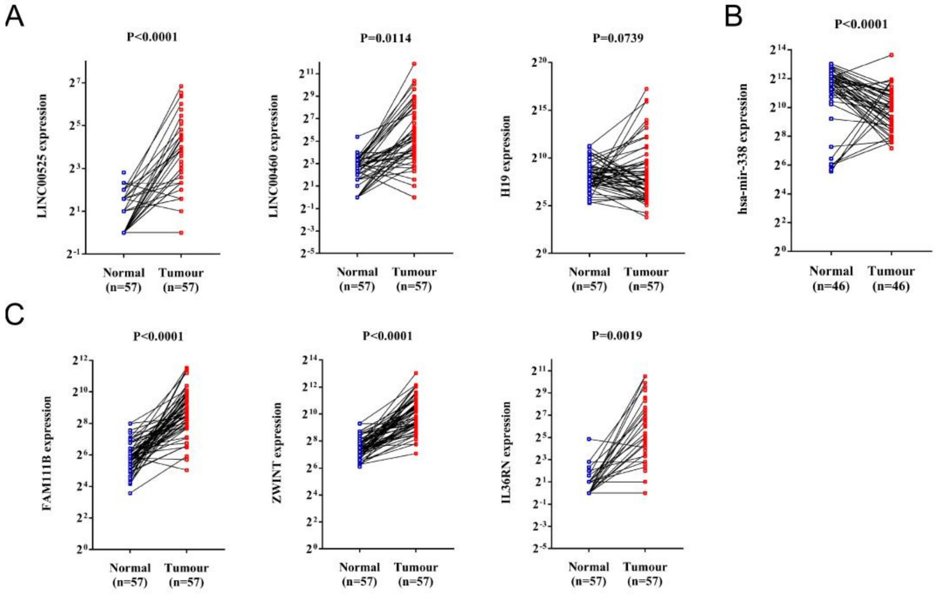

Figure S5. Pairing of gene expression patterns between tumor tissue and normal tissue in patients with LUAD. (A) DElncRNA. (B) DEmiRNA. (C) DEmRNA.

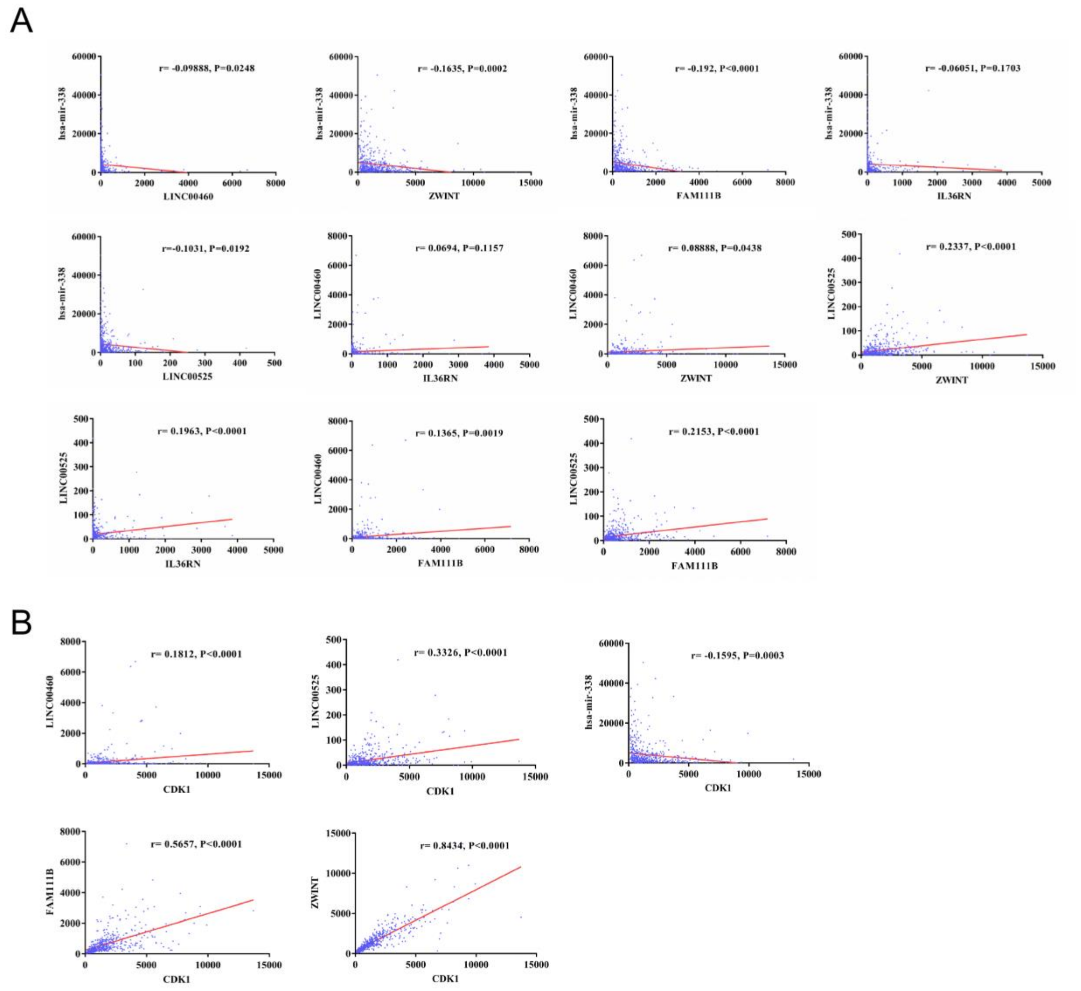

Figure S6. Pairwise analysis of expression between genes. (A) Pairwise analysis between DERNAs. (B) Pairwise analysis between DERNAs and CDK1.

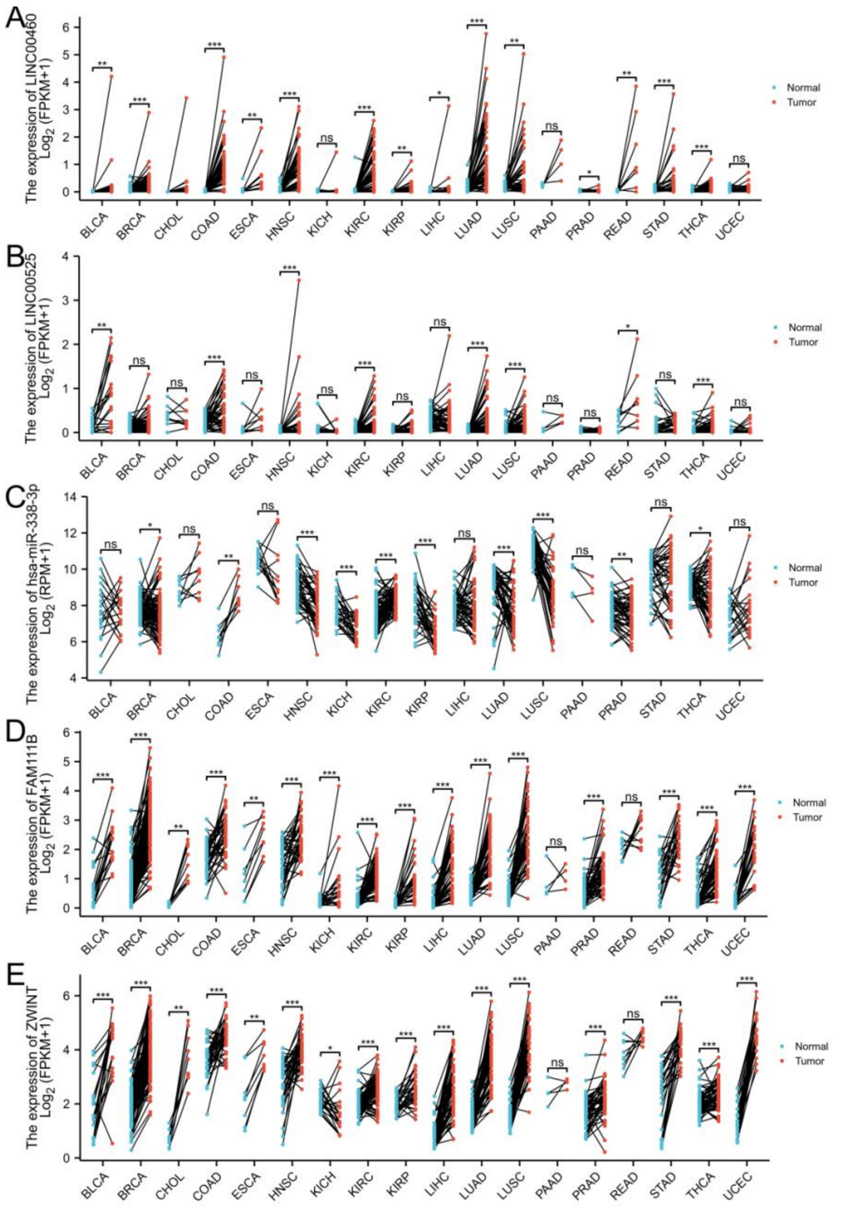

Figure S7. DERNAs expression in pan-cancer. Significance markers: ns, $p \geq 0.05$; *, $p < 0.05$; **, $p < 0.01$; ***, $p < 0.001$.

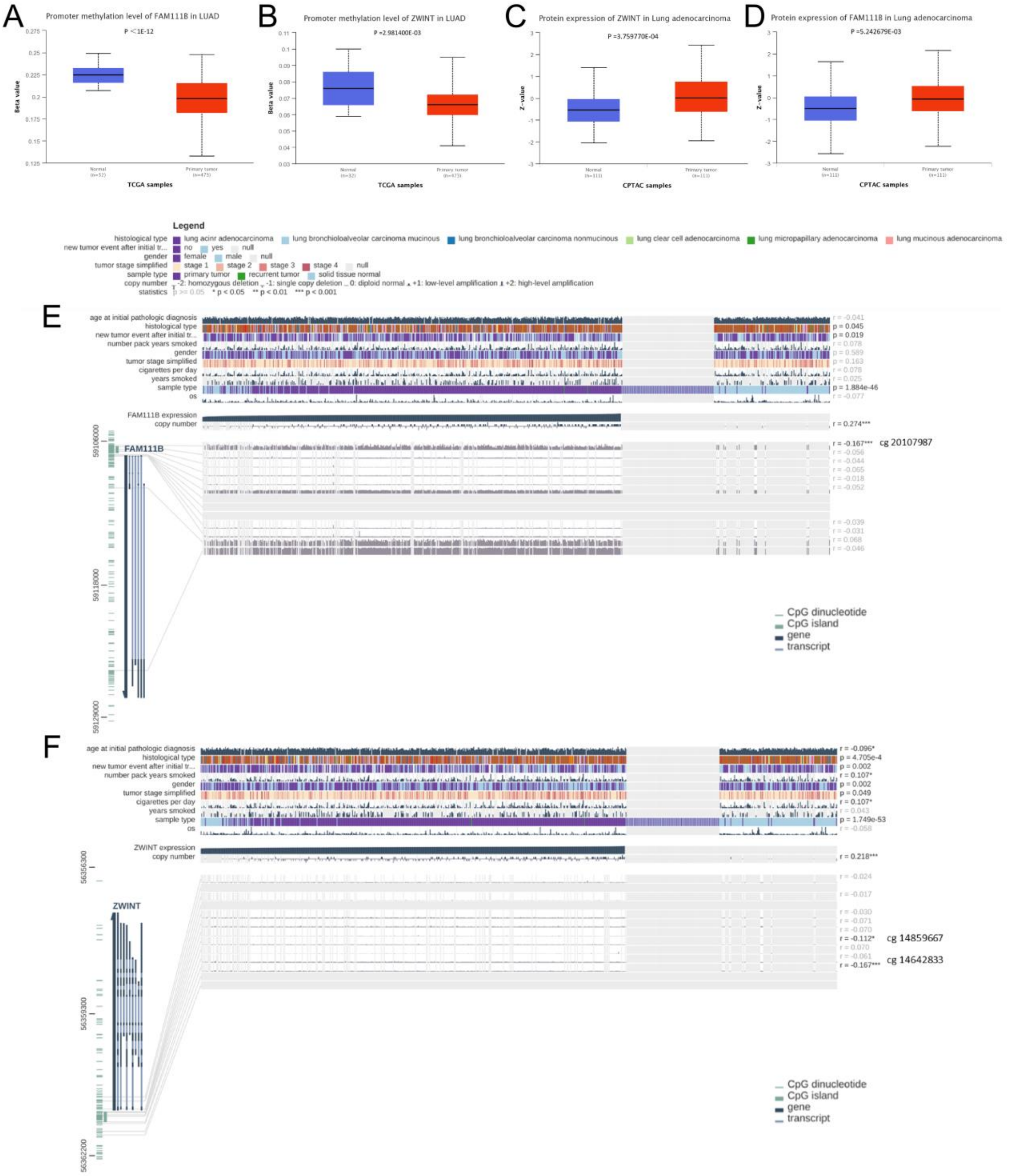

Figure S8. Methylation and phosphorylation analysis of FAM111B and ZWINT. (A-D) Methylation and Phosphorylation levels of FAM111B and ZWINT. (E-F) Relationship between methylation sites and expression of (E) FAM111B and (F) ZWINT. Significance markers: ns, $p \geq 0.05$; *, $p < 0.05$; **, $p < 0.01$; ***, $p < 0.001$.

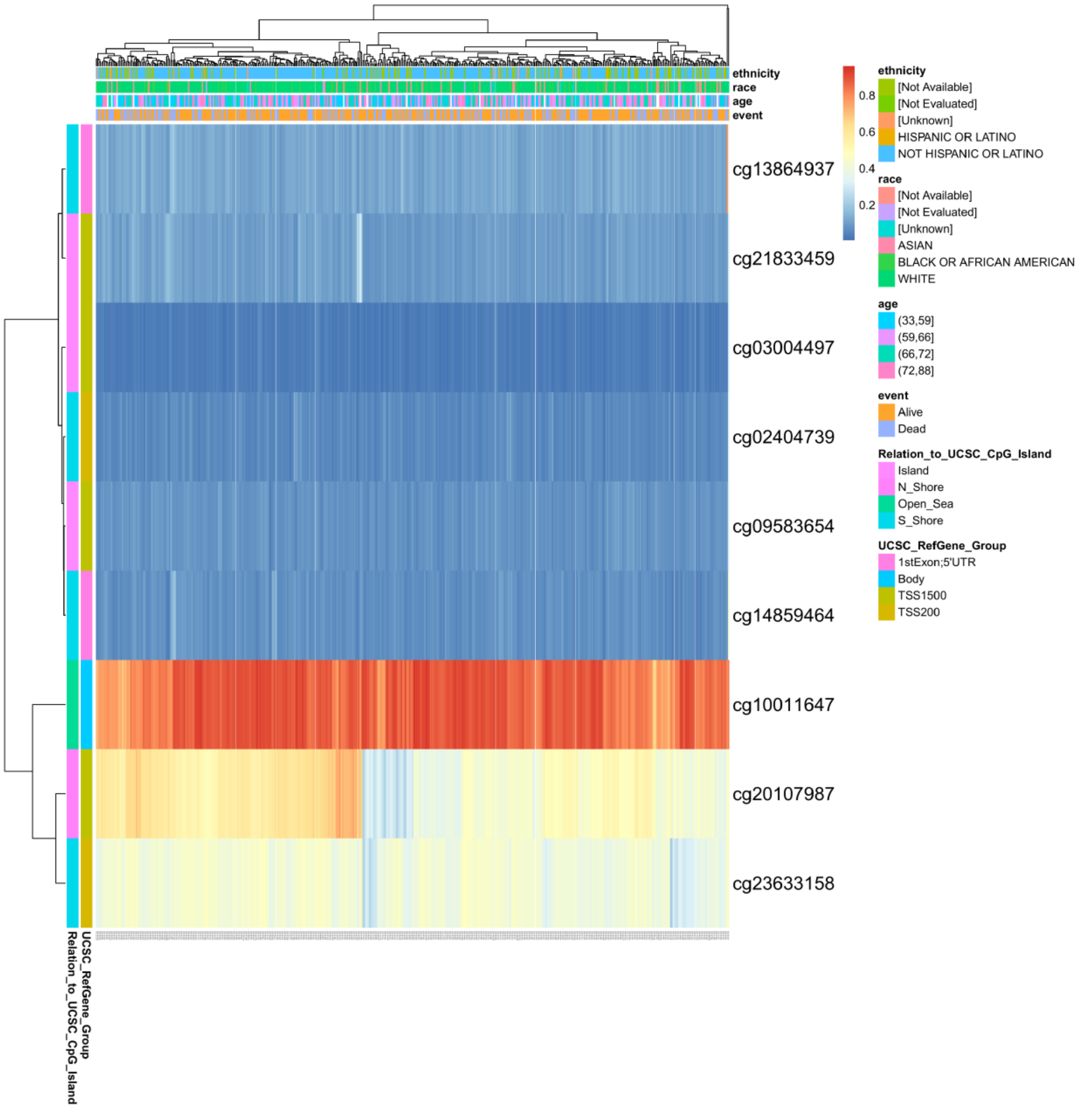

Figure S9. Distribution of different methylation regions related to FAM111B.

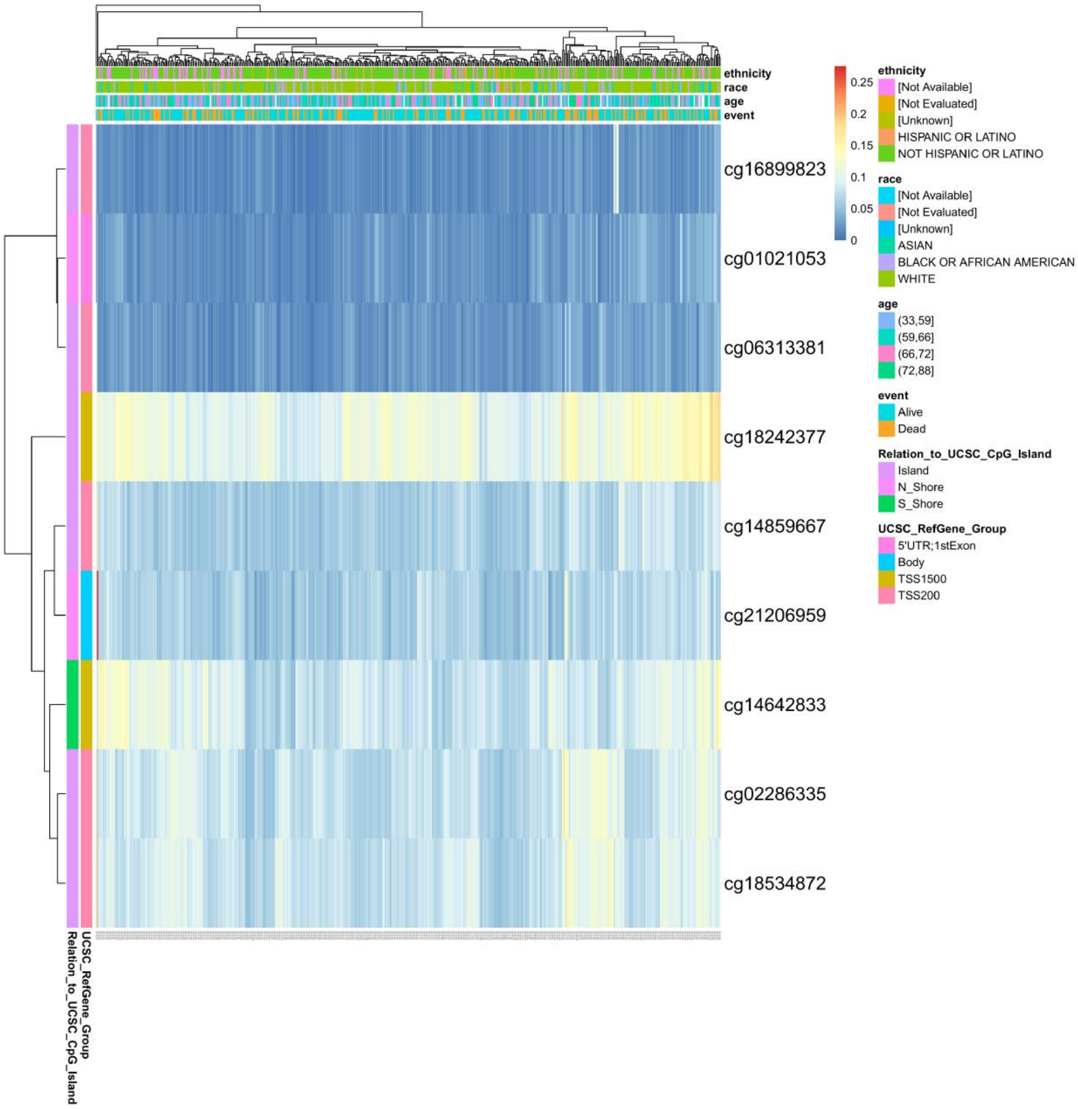

Figure S10. Distribution of different methylation regions related to ZWINT.

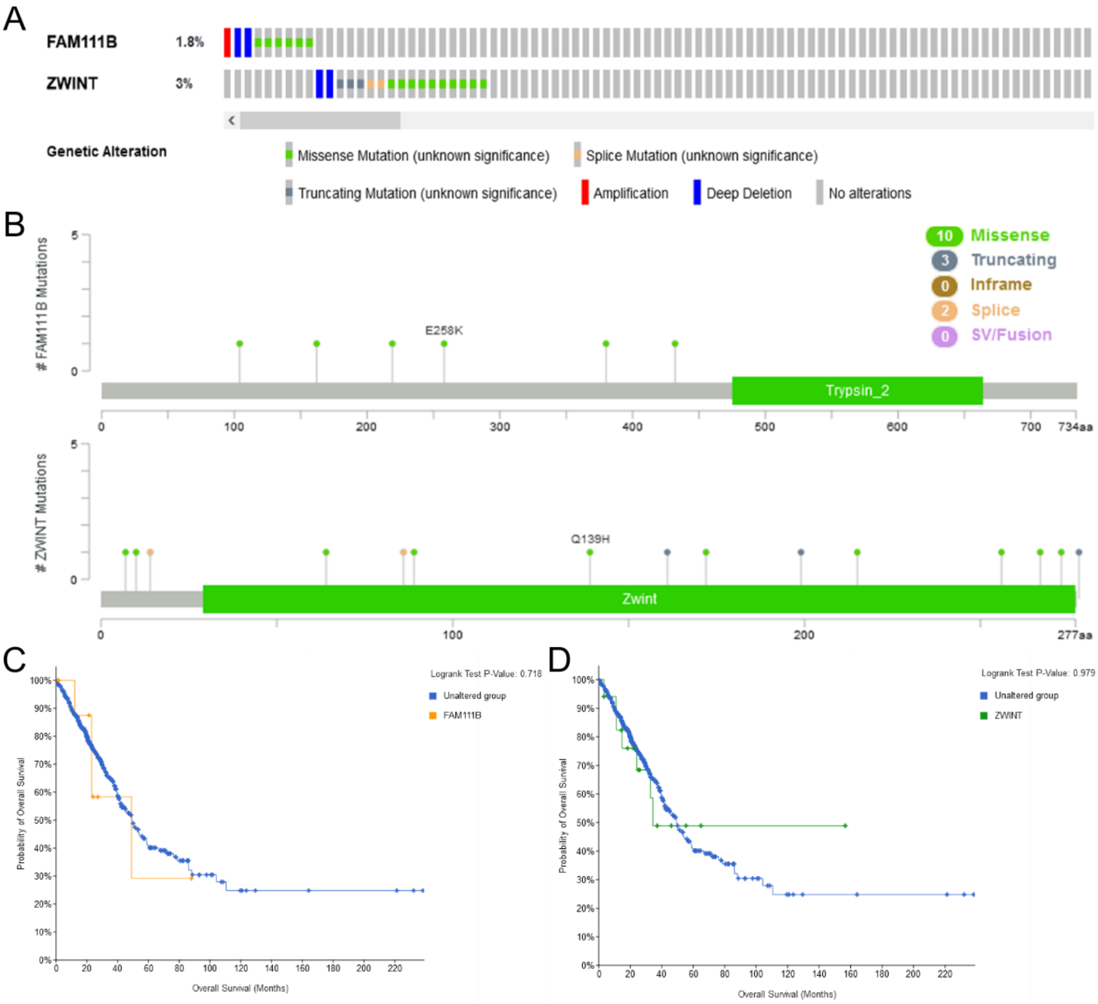

Figure S11. Mutations of FAM111B and ZWINT in LUAD. (A) Frequency and type of mutations in LUAD. (B) Mutation sites of FAM111B and ZWINT. (C) Correlation between mutations of FAM111B and patient OS. (D) Correlation between mutations of FAM111B and patient OS.

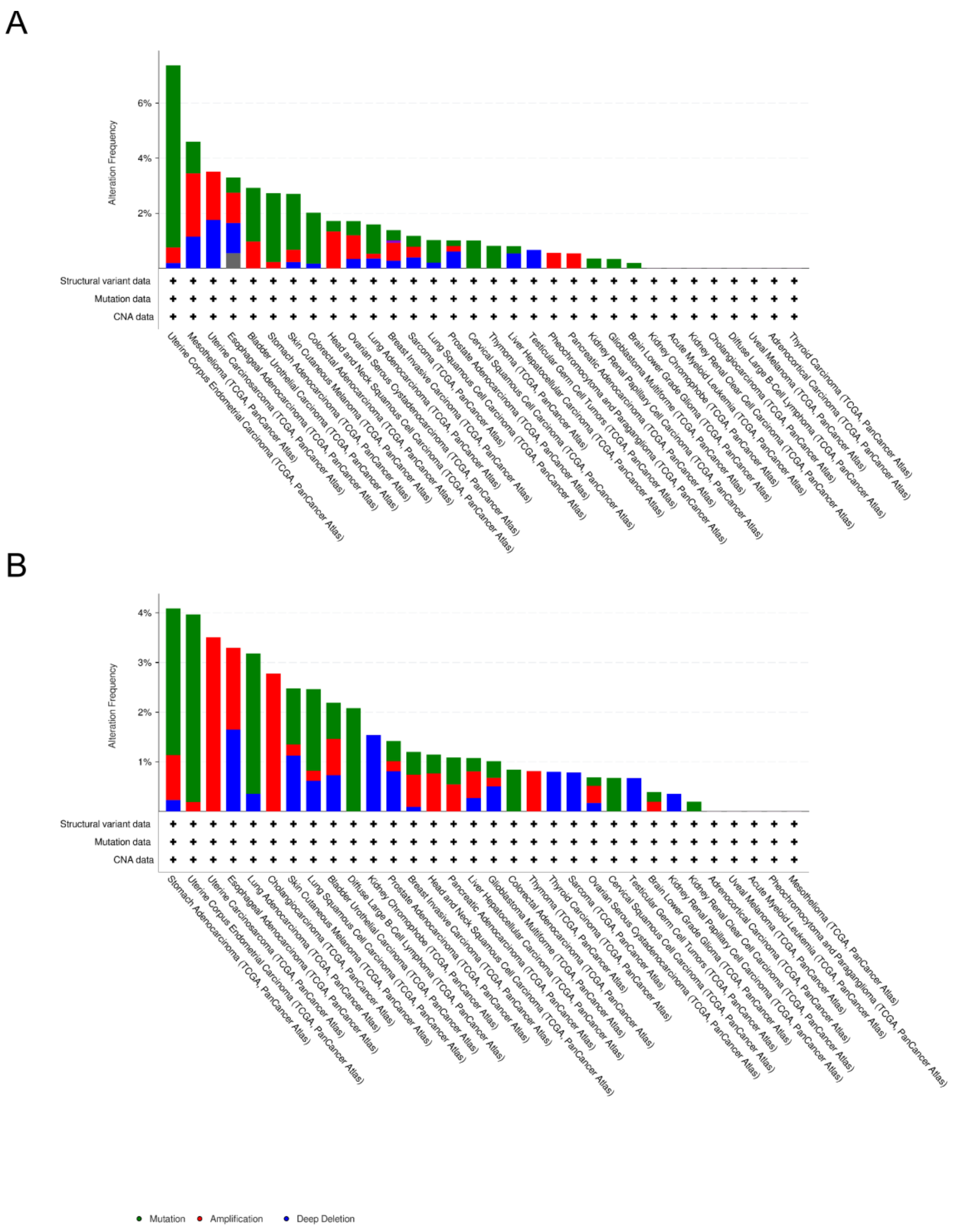

Figure S12. Frequency and type of mutations of FAM111B and ZWINT genes in pan-cancer.

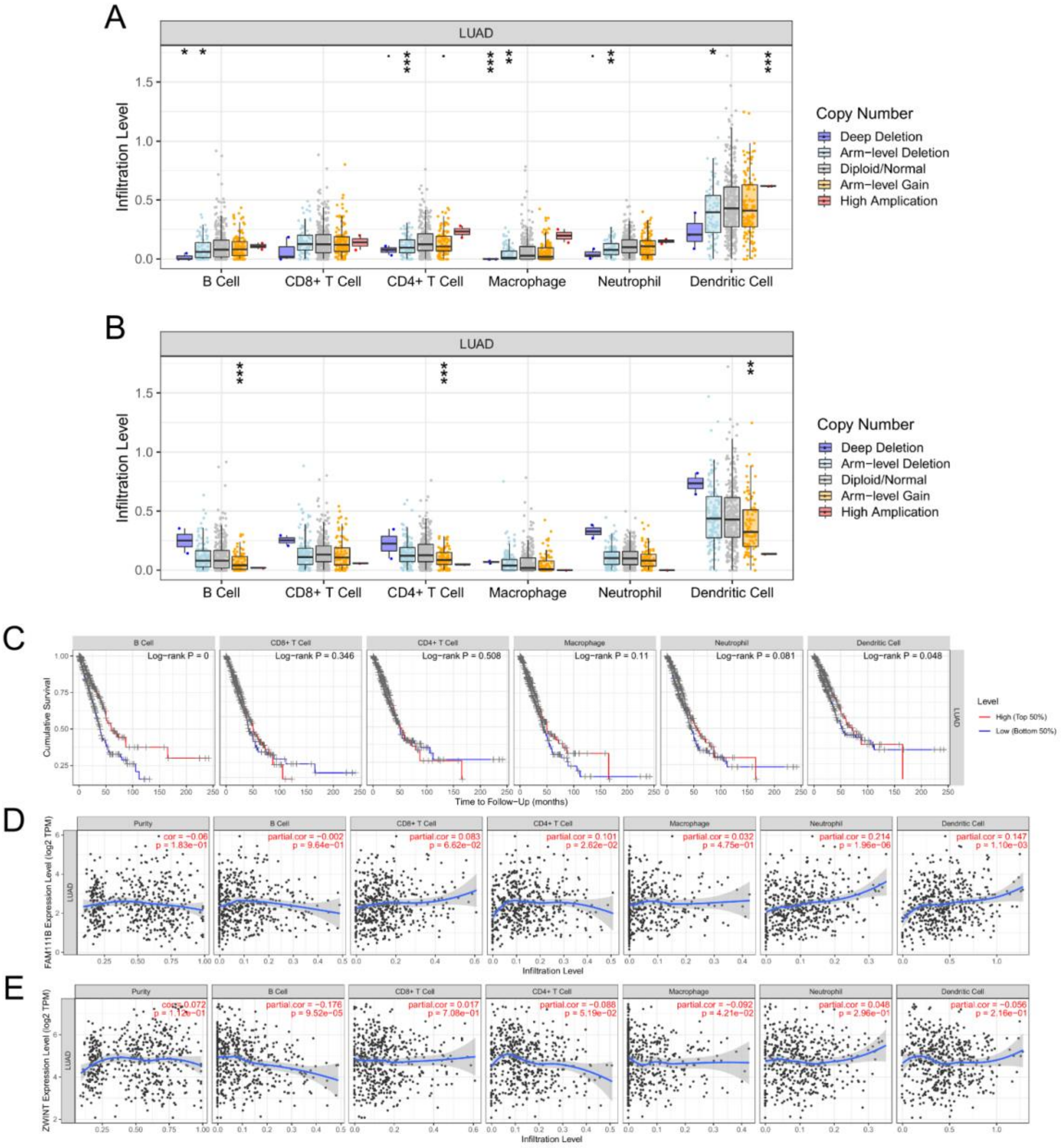

Figure S13. Immune infiltration levels of FAM111B and ZWINT. Significance markers: ns, $p \geqslant 0.05$; *, $p < 0.05$; **, $p < 0.01$; ***, $p < 0.001$. (A and B) Gene copy number of (A) FAM111B and (B) ZWINT in relation to the level of immune cell tumor infiltration in LUAD. (C) Correlation of immune cells with patient OS. (D and E) Correlation of (D) FAM111B and (E) ZWINT expression with the level of immune infiltration in LUAD.

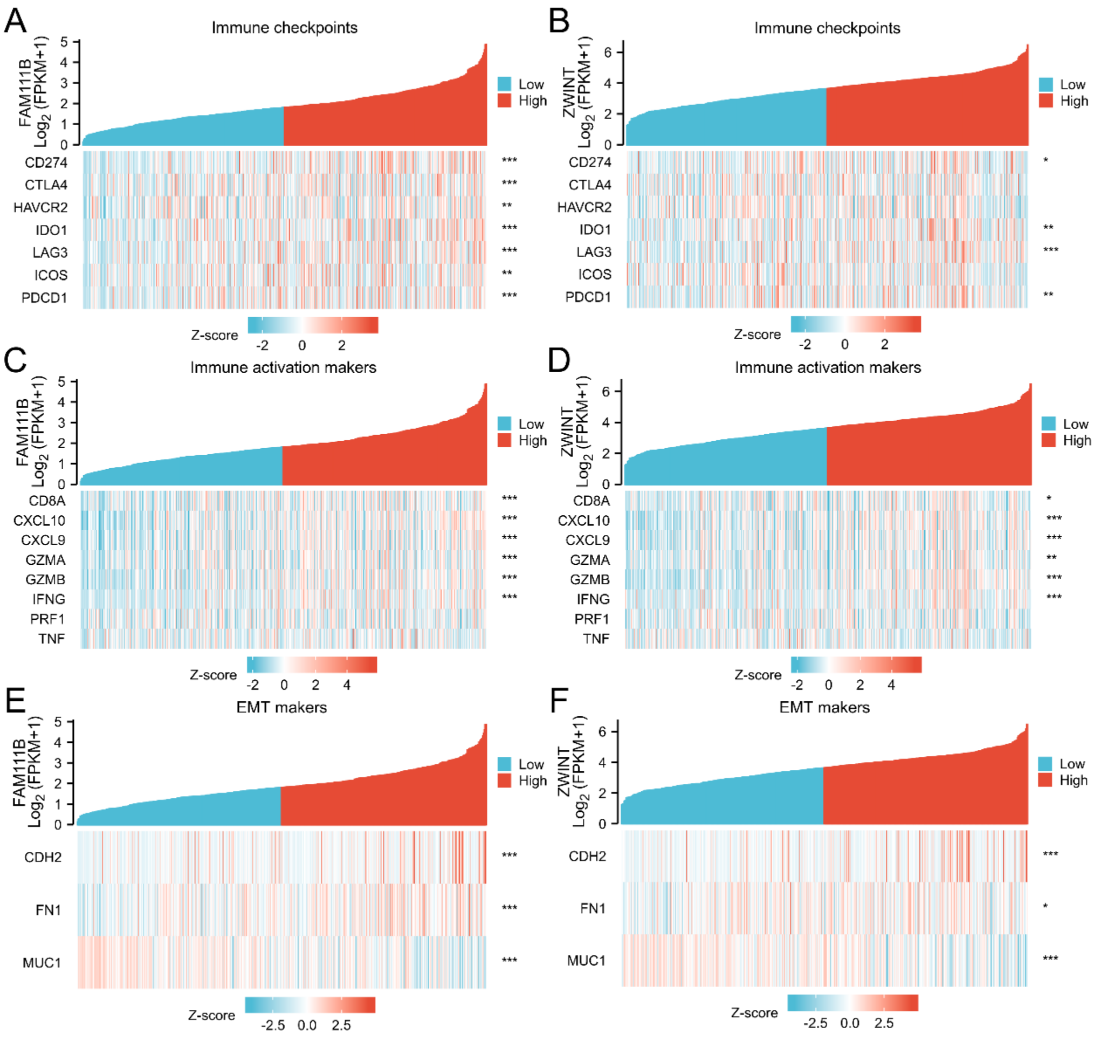

Figure S14. The expressions of FAM111B and ZWINT were associated with immune checkpoints, immune activation makers and EMT makers. Significance markers: ns, $p \geq 0.05$; *, $p < 0.05$; **, $p < 0.01$; ***, $p < 0.001$.

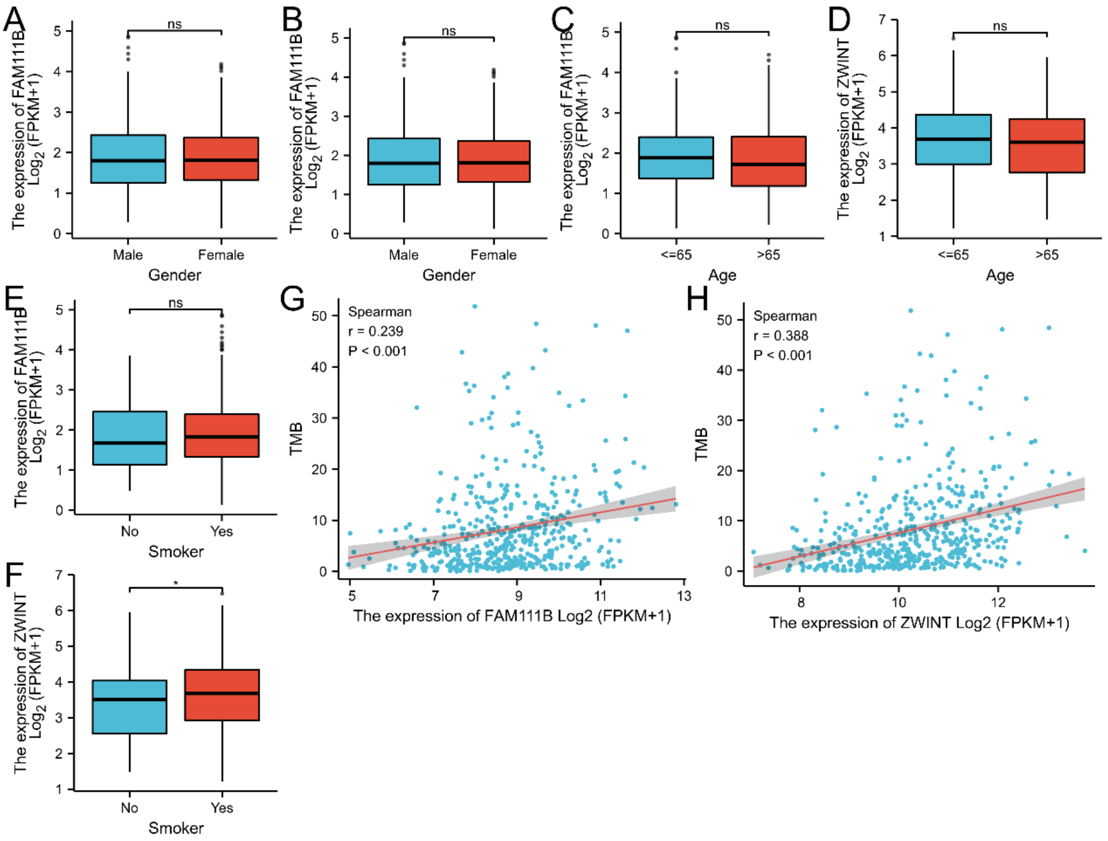

Figure S15. Correlation between expression of FAM111B and ZWINT and clinical information and TMB. Significance markers: ns, $p \geq 0.05$; *, $p < 0.05$; **, $p < 0.01$; ***, $p < 0.001$. (A and B) Correlation between FAM111B and ZWINT expression and gender. (C and D) Correlation between FAM111B and ZWINT expression and age. (E and F) Correlation between FAM111B and ZWINT expression and smoker. (G and H) Correlation between FAM111B and ZWINT expression and TMB.

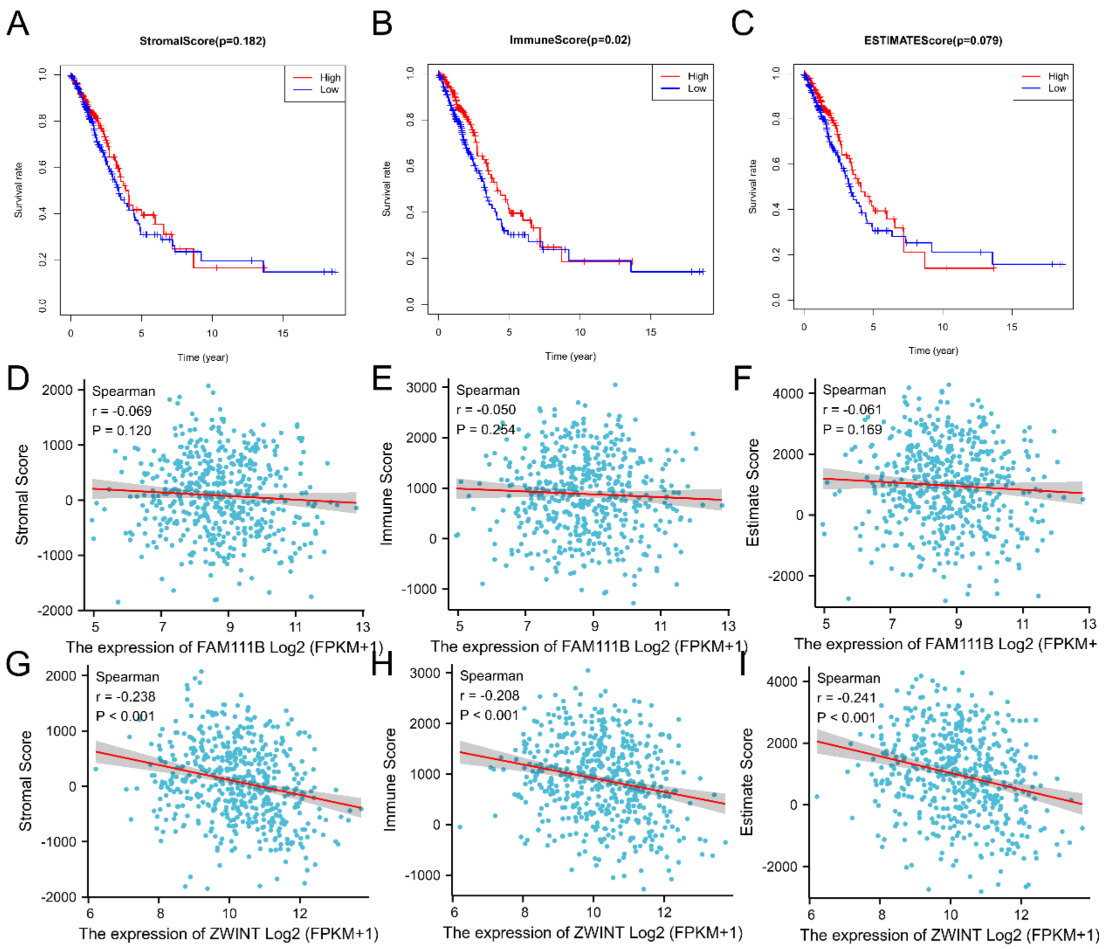

Figure S16. Comparison between TIME and OS and FAM111B/ZWINT expression of patients. (A-C) The correlation between OS of patients and stromal score, immune score and estimate score. (D-E) Correlation between the expression of FAM111B and stromal score, immune score and estimate score. (G-I) Correlation between the expression of ZWINT and stromal score, immune score and estimate score.

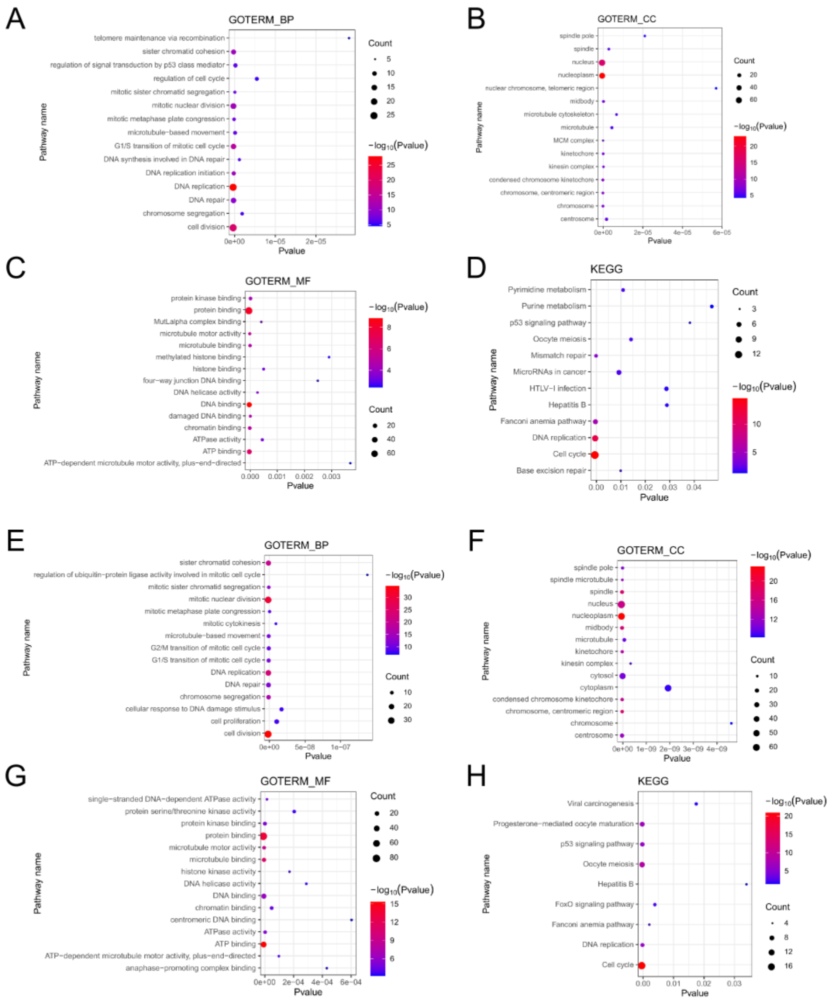

Figure S17. GO and KEGG enrichment analysis. (A-H) Enrichment analysis of (A-D) FAM111B and (E-H) ZWINT.

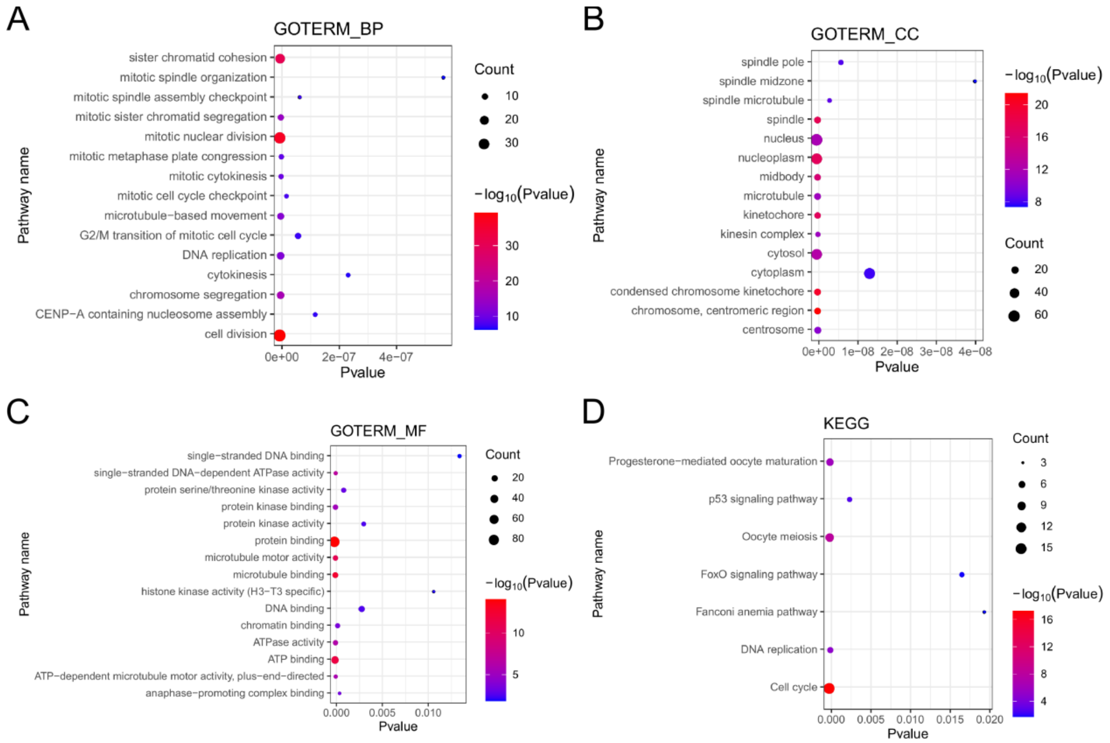

Figure S18. Enrichment analysis of CDK1.

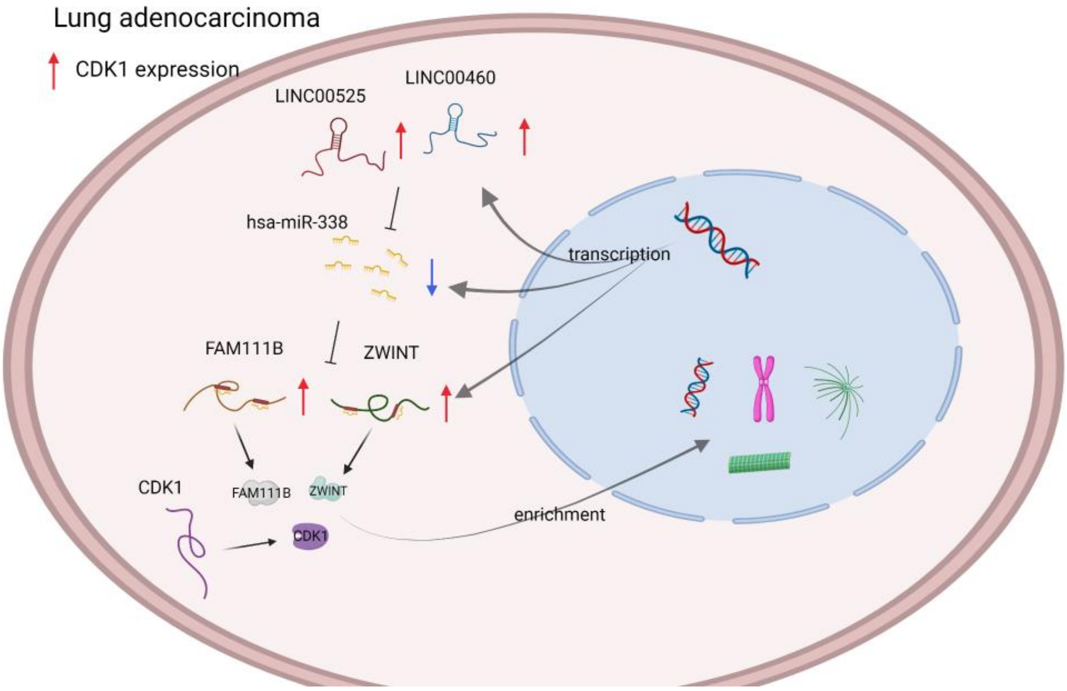

Figure S19. Mutual regulation between genes

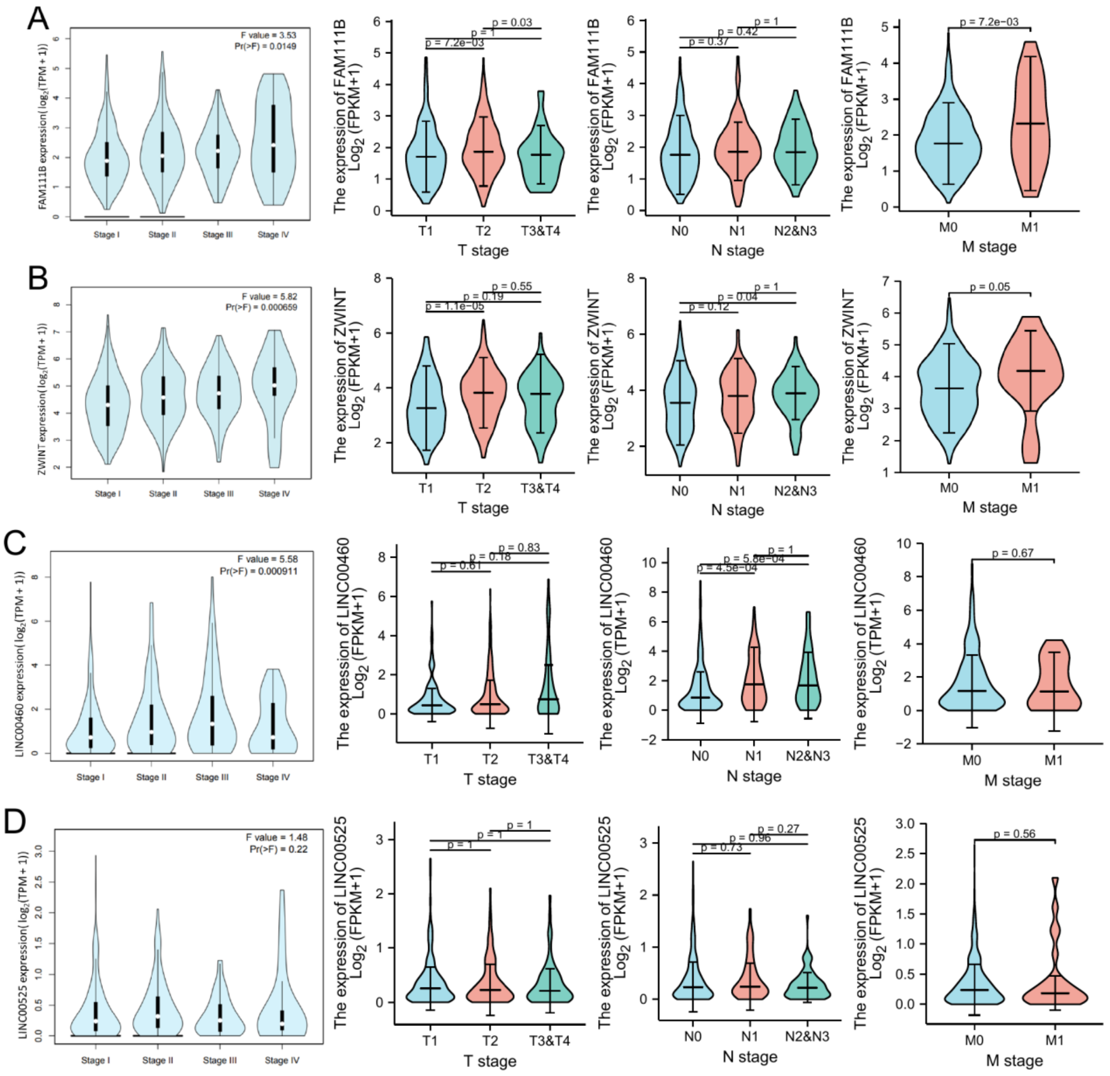

Figure S20. Correlation between gene expression and LUAD TNM stage.

Supplementary Table

Table S1. List of primers for RT-qPCR.

Primer	Primer sequence (5′-3′)
GAPDH-Forward	CAGGAGGCATTGCTGATGAT
GAPDH-Reverse	GAAGGCTGGTCATTT
FAM111B-Forward	AGACACATGCTGACACACCTGTTG
FAM111B-Reverse	GGGCTGTTTCATGCTTGTTGACTTC
ZWINT-Forward	GCAGAGCAGTCCAGAACCAGTG
ZWINT-Reverse	GCCTCAGGGAACAACAGCTTACC
U6-Forward	AGAGAAGATTAGCATGGCCCCTG
U6-Reverse	CAGTGCAGGGTCCGAGGT
U6-RT Primer	GTCGTATCCAGTGCAGGGTCCGAGGTATTCGCACTGGATACGACAAAATA
has-mir-338-Forward	CGCGTCCAGCATCAGTGATT
has-mir-338-Reverse	AGTGCAGGGTCCGAGGTATT
has-mir-338-RT Primer	GTCGTATCCAGTGCAGGGTCCGAGGTATTCGCACTGGATACGACCAACAA
LINC00460-Forward	TCGGCTAAGAGTCACCCTGGATG
LINC00460-Reverse	CACAGACGCCTCCCACACAATG
LINC00525-Forward	AGACCCTATGACTCCCGCACATG
LINC00525-Reverse	GCTTGCCTGCTGCCTGCTATAG

Table S2. Mann-Whitney U test was used to compare the expression of CDK1 in normal tissues and pan-cancerous tissues.

Types of cancer		Sample size	Minimum	Maximum	Median	Interquartile range	P value
ACC	Tumor	79	0.181	5.274	2.627	2.233	
BLCA	Normal	19	0.159	3.441	1.104	1.759	< 0.001
BLCA	Tumor	414	0.588	5.914	3.965	1.109	
BRCA	Normal	113	0.271	3.286	1.094	0.579	< 0.001
BRCA	Tumor	1109	0.401	7.087	3.469	1.374	
CESC	Normal	3	0.612	1.37	0.971	0.379	0.003
CESC	Tumor	306	2.707	6.894	4.688	0.747	
CHOL	Normal	9	0.109	0.405	0.171	0.147	< 0.001
CHOL	Tumor	36	0.539	4.177	2.334	0.702	
COAD	Normal	41	0.729	3.548	2.622	0.723	< 0.001
COAD	Tumor	480	1.172	6.386	4.141	0.925	
DLBC	Tumor	48	2.448	5.49	4.222	0.806	
ESCA	Normal	11	0.514	3.45	1.881	1.606	< 0.001
ESCA	Tumor	162	1.538	5.658	4.02	0.983	
GBM	Normal	5	0.237	0.509	0.271	0.024	< 0.001
GBM	Tumor	169	0.684	5.043	3.023	1.175	
HNSC	Normal	44	0.259	3.53	2.609	1.188	< 0.001
HNSC	Tumor	502	1.22	6.067	3.741	1.009	
KICH	Normal	24	0.68	1.956	1.126	0.503	0.004
KICH	Tumor	65	0.327	3.476	0.818	0.472	
KIRC	Normal	72	0.65	3.845	1.066	0.467	< 0.001
KIRC	Tumor	539	0.375	4.986	1.49	0.672	
KIRP	Normal	32	0.45	1.583	0.826	0.42	0.132
KIRP	Tumor	289	0.153	4.653	0.895	0.679	
LAML	Tumor	151	0.487	5.217	3.019	1.19	
LGG	Tumor	529	0.101	5.346	1.248	1.254	
LIHC	Normal	50	0	1.094	0.218	0.177	< 0.001
LIHC	Tumor	374	0.152	5.508	1.907	1.669	
LUAD	Normal	59	0.83	2.102	1.236	0.401	< 0.001
LUAD	Tumor	535	0.492	6.092	3.005	1.543	
LUSC	Normal	49	0.699	2.535	1.209	0.437	< 0.001
LUSC	Tumor	502	1.164	6.067	4.154	0.952	
MESO	Tumor	86	0.602	4.46	2.585	1.131	
OV	Tumor	379	0.875	5.736	3.827	1.031	
PAAD	Normal	4	1.336	2.503	1.558	0.377	0.051
PAAD	Tumor	178	0.183	4.238	2.445	0.978	
PCPG	Normal	3	0.302	0.405	0.327	0.051	0.007
PCPG	Tumor	183	0.217	2.965	0.956	0.741	

Table S2 | continued

Types of cancer		Sample size	Minimum	Maximum	Median	Interquartile range	P value
PRAD	Normal	52	0.293	2.874	0.797	0.583	< 0.001
PRAD	Tumor	499	0.242	5.545	1.275	0.927	
READ	Normal	10	2.197	3.853	2.938	0.537	< 0.001
READ	Tumor	167	1.111	5.505	4.035	0.858	
SARC	Normal	2	0.807	1.307	1.057	0.25	< 0.001
SARC	Tumor	263	0.684	5.499	3.373	1.434	
SKCM	Normal	1	3.195	3.195	3.195	0	NA
SKCM	Tumor	471	0.311	5.576	2.988	1.085	
STAD	Normal	32	0.194	3.445	1.796	1.866	< 0.001
STAD	Tumor	375	0.574	6.183	3.659	1.045	
TGCT	Tumor	156	1.56	5.088	3.541	0.998	
THCA	Normal	58	0.455	2.986	0.953	0.451	< 0.001
THCA	Tumor	510	0.256	3.008	1.197	0.513	
THYM	Normal	2	2.957	4.934	3.946	0.988	NA
THYM	Tumor	119	0.878	5.646	4.197	1.525	
UCEC	Normal	35	0.231	3.932	1.122	0.679	< 0.001
UCEC	Tumor	552	0.532	6.385	3.791	1.129	
UCS	Tumor	56	3.249	5.956	4.267	0.698	
UVM	Tumor	80	0.173	3.097	1.165	0.8	

Table S3. Wilcoxon signed rank test was used to compare the expression of CDK1 in adjacent and pan-cancerous tissues.

Types of cancer		Sample size	Minimum	Maximum	Median	Interquartile range	P value
BLCA	Normal	19	0.159	3.441	1.104	1.759	< 0.001
BLCA	Tumor	19	1.752	5.802	4.21	1.052	
BRCA	Normal	112	0.271	3.286	1.086	0.585	< 0.001
BRCA	Tumor	112	0.756	5.987	3.449	1.512	
CHOL	Normal	9	0.109	0.405	0.171	0.147	0.004
CHOL	Tumor	9	1.298	4.07	2.544	1.29	
COAD	Normal	41	0.729	3.548	2.622	0.723	< 0.001
COAD	Tumor	41	1.486	5.003	3.961	1.204	
ESCA	Normal	8	0.514	3.115	1.901	1.178	0.008
ESCA	Tumor	8	2.875	4.313	3.573	0.326	
HNSC	Normal	43	0.259	3.53	2.632	1.195	< 0.001
HNSC	Tumor	43	2.477	4.495	3.489	0.732	
KICH	Normal	23	0.68	1.956	1.147	0.514	0.393
KICH	Tumor	23	0.379	3.476	0.782	0.848	
KIRC	Normal	72	0.65	3.845	1.066	0.467	< 0.001
KIRC	Tumor	72	0.613	3.403	1.488	0.703	
KIRP	Normal	31	0.45	1.583	0.813	0.43	0.018
KIRP	Tumor	31	0.153	3.61	1.031	1.319	
LIHC	Normal	50	0	1.094	0.218	0.177	< 0.001
LIHC	Tumor	50	0.355	4.41	1.727	1.203	
LUAD	Normal	57	0.83	2.102	1.236	0.381	< 0.001
LUAD	Tumor	57	0.883	6.092	2.852	1.462	
LUSC	Normal	49	0.699	2.535	1.209	0.437	< 0.001
LUSC	Tumor	49	1.194	5.488	4.04	1.155	
PAAD	Normal	4	1.336	2.503	1.558	0.377	0.25
PAAD	Tumor	4	1.881	2.417	2.267	0.236	
PRAD	Normal	52	0.293	2.874	0.797	0.583	< 0.001
PRAD	Tumor	52	0.392	5.545	1.203	0.59	
READ	Normal	9	2.197	3.853	2.878	0.575	0.008
READ	Tumor	9	3.433	4.7	4.011	0.453	
STAD	Normal	27	0.194	3.445	1.771	1.942	< 0.001
STAD	Tumor	27	1.993	6.183	3.567	1.13	
THCA	Normal	58	0.455	2.986	0.953	0.451	0.011
THCA	Tumor	58	0.607	3.008	1.203	0.521	
UCEC	Normal	23	0.231	2.565	0.873	0.589	< 0.001
UCEC	Tumor	23	1.901	4.999	4.116	0.782	

Table S4. Co-mutation analysis of mutations of FAM111B and ZWINT and mutations of common genes in lung cancer.

A	B	Neither	A Not B	B Not A	Both	Log2 Odds Ratio	P Value	Tendency
EGFR	KRAS	1177	1062	881	32	<-3	<0.001	Mutual exclusivity
TP53	LRP1B	494	299	74	154	1.782	<0.001	Co-occurrence
KRAS	ERBB2	2079	902	160	11	-2.658	<0.001	Mutual exclusivity
KRAS	TP53	1119	586	1120	327	-0.843	<0.001	Mutual exclusivity
EGFR	LRP1B	524	269	202	26	-1.996	<0.001	Mutual exclusivity
EGFR	TP53	1200	505	858	589	0.706	<0.001	Co-occurrence
KRAS	LRP1B	617	176	128	100	1.454	<0.001	Co-occurrence
LRP1B	FAT4	599	160	48	52	2.02	<0.001	Co-occurrence
EGFR	ALK	1918	1070	140	24	-1.702	<0.001	Mutual exclusivity
EGFR	BRAF	1893	1060	165	34	-1.442	<0.001	Mutual exclusivity
EGFR	KMT2D	1781	1035	145	29	-1.539	<0.001	Mutual exclusivity
EGFR	RET	1961	1080	97	14	-1.932	<0.001	Mutual exclusivity
EGFR	MET	1897	1058	161	36	-1.319	<0.001	Mutual exclusivity
TP53	KMT2D	1543	1273	64	110	1.059	<0.001	Co-occurrence
KRAS	BRAF	2071	882	168	31	-1.207	<0.001	Mutual exclusivity
KRAS	MET	2073	882	166	31	-1.188	<0.001	Mutual exclusivity
TP53	FAT4	436	323	34	66	1.39	<0.001	Co-occurrence
TP53	ERBB2	1640	1341	65	106	0.996	<0.001	Co-occurrence
RET	LRP1B	780	13	211	17	2.273	<0.001	Co-occurrence
TP53	APC	1639	1350	66	97	0.835	0.001	Co-occurrence
LRP1B	ALK	765	206	28	22	1.545	0.002	Co-occurrence
KMT2D	ALK	2675	154	141	20	1.301	0.004	Co-occurrence
LRP1B	KMT2D	619	189	28	23	1.428	0.004	Co-occurrence
EGFR	ERBB2	1928	1053	130	41	-0.792	0.006	Mutual exclusivity
FAT4	ALK	725	87	34	13	1.672	0.008	Co-occurrence
EGFR	FAT4	513	246	81	19	-1.032	0.016	Mutual exclusivity
BRAF	APC	2809	180	144	19	1.042	0.027	Co-occurrence
LRP1B	APC	750	204	43	24	1.037	0.028	Co-occurrence
RET	FAT4	740	19	92	8	1.76	0.036	Co-occurrence
BRAF	LRP1B	751	42	207	21	0.859	0.103	
RET	PIK3CA	2848	109	193	2	-1.885	0.105	
KRAS	KMT2D	2024	792	113	61	0.464	0.119	
KRAS	FAT4	576	183	67	33	0.633	0.138	
ZWINT	EGFR	581	13	264	1	-2.562	0.142	
ZWINT	KMT2D	797	11	48	3	2.179	0.155	
FAT4	KMT2D	718	90	41	10	0.96	0.204	
EGFR	PIK3CA	1941	1016	117	78	0.349	0.21	
ZWINT	RET	820	12	25	2	2.451	0.216	
RET	BRAF	2845	108	196	3	-1.31	0.216	

Table S4 | continued

A	B	Neither	A Not B	B Not A	Both	Log_2 Odds Ratio	P Value	Tendency
FAM111B	ERBB2	810	8	39	2	2.376	0.23	
TP53	RET	1653	1388	52	59	0.434	0.216	
APC	KMT2D	2677	139	161	13	0.637	0.284	
ERBB2	PIK3CA	2801	156	180	15	0.581	0.284	
FAM111B	TP53	467	3	382	7	1.512	0.284	
FAM111B	KMT2D	800	8	49	2	2.029	0.306	
TP53	PIK3CA	1608	1349	97	98	0.268	0.309	
TACSTD2	FAT4	751	8	97	3	1.538	0.317	
KRAS	PIK3CA	2093	864	146	49	-0.299	0.317	
FAM111B	EGFR	585	9	264	1	-2.022	0.331	
APC	ALK	2837	151	152	12	0.569	0.331	

Table S5. TIMER database query for correlation of FAM111B and ZWINT genes with 16 markers of immune cells infiltrating tumors.

Description	Gene markers	LUAD-FAM111B		LUAD-ZWINT	
		Correlation	Pvalue	Correlation	Pvalue
CD8+ T cell	CD8A	0.179	4.66E-05	0.104	1.85E-02
	CD8B	0.163	2.06E-04	0.121	6.08E-03
T cell	CD3D	0.062	1.57E-01	-0.044	3.22E-01
	CD3E	0.067	1.31E-01	-0.075	9.06E-02
	CD2	0.082	6.16E-02	-0.065	1.39E-01
B cell	CD19	-0.025	5.74E-01	0.117	7.61E-03
	CD79A	-0.037	3.96E-01	-0.135	2.10E-03
Monocyte	CD86	0.187	2.02E-05	0.011	8.09E-01
	CD115 (CSF1R)	0.162	2.17E-04	-0.085	5.33E-02
TAM	CCL2	0.214	1.07E-06	0.035	4.28E-01
	CD68	0.125	4.46E-03	0.003	9.47E-01
	IL10	0.117	7.72E-03	-0.016	7.16E-01
M1 Macrophage	INOS (NOS2)	0.051	2.47E-01	0.061	1.70E-01
	IRF5	0.168	1.34E-04	0.029	5.13E-01
	COX2 (PTGS2)	0.123	5.14E-03	0.075	8.92E-02
M2 Macrophage	CD163	0.216	8.34E-07	0.051	2.44E-01
	VSIG4	0.069	1.15E-01	-0.065	1.38E-01
	MS4A4A	0.07	1.12E-01	-0.081	6.50E-02
Neutrophils	CD66b (CEACAM8)	-0.181	3.65E-05	-0.319	1.20E-13
	CD11b (ITGAM)	0.13	3.06E-06	-0.107	1.55E-02
	CCR7	-0.044	3.15E-01	-0.197	7.19E-06
Natural killer cell	KIR2DL1	-0.03	4.90E-01	0.045	3.04E-01
	KIR2DL3	0.137	1.86E-03	0.159	2.90E-04
	KIR2DL4	0.289	2.40E-11	0.333	8.37E-15
	KIR3DL1	0.025	5.69E-01	0.041	3.53E-01
	KIR3DL2	0.118	7.29E-03	0.129	3.46E-03
	KIR3DL3	0.073	9.84E-02	0.158	3.18E-04
	KIR2DS4	0.055	2.09E-01	0.07	1.11E-01
Dendritic cell	HLA-DPB1	-0.119	6.95E-03	-0.357	6.25E-17
	HLA-DQB1	-0.057	2.00E-01	-0.278	1.26E-10
	HLA-DRA	-0.072	1.05E-01	-0.299	5.77E-12
	HLA-DPA1	-0.035	4.23E-01	-0.289	2.83E-11
	BDCA-1 (CD1C)	-0.242	2.78E-08	-0.475	2.27E-30
	BDCA-4 (NRP1)	0.111	1.17E-02	-0.041	3.50E-01
	CD11c (ITGAX)	0.154	4.52E-04	-0.024	5.93E-01

Table S5 | continued

Description	Gene markers	LUAD-FAM111B		LUAD-ZWINT	
		Correlation	Pvalue	Correlation	Pvalue
Th1	T-bet (TBX21)	0.132	2.69E-03	0.013	7.62E-01
	STAT4	0.128	3.50E-03	-0.027	5.45E-01
	STAT1	0.488	0.00E+00	0.383	0.00E+00
	IFN-γ (IFNG)	0.266	8.29E-10	0.258	2.83E-09
	TNF-α (TNF)	0.112	1.11E-02	-0.033	4.62E-01
Th2	GATA3	0.211	1.41E-06	0.019	6.73E-01
	STAT6	-0.106	1.59E-02	-0.182	3.48E-05
	STAT5A	0.146	8.84E-04	-0.068	1.21E-01
	IL13	0.033	4.52E-01	-0.016	7.15E-01
Tfh	BCL6	0.022	6.16E-01	-0.103	1.94E-02
	IL21	0.192	1.15E-05	0.156	3.70E-04
Th17	STAT3	0.127	3.92E-03	0	9.98E-01
	IL17A	0.076	8.63E-02	0.091	3.86E-02
Treg	FOXP3	0.212	1.30E-06	0.057	1.93E-01
	CCR8	0.213	1.05E-06	0.059	1.79E-01
	STAT5B	0.13	3.18E-03	-0.012	7.88E-01
	TGFβ (TGFB1)	0.049	2.66E-01	-0.157	3.56E-04
T cell exhaustion	PD-1 (PDCD1)	0.221	4.14E-07	0.136	1.93E-03
	CTLA4	0.175	6.41E-05	0.078	7.88E-02
	LAG3	0.305	2.05E-12	0.221	4.41E-07
	TIM-3 (HAVCR2)	0.171	9.98E-05	-0.002	9.64E-01
	GZMB	0.309	7.97E-13	0.336	5.07E-15

Table S6. GEPIA database verifies the correlation between FAM111B and immune cell markers.

Description	Gene markers	LUAD-FAM111B	
		Cor	Pvalue
CD8+ T cell	CD8A	0.12	0.01
Monocyte	CD86	0.17	0.00012
TAM	CCL2	0.12	0.0083
M2 Macrophage	CD163	0.11	0.019
Neutrophils	CD66b (CEACAM8)	-0.1	0.029
	CD11b (ITGAM)	0.11	0.02
Natural killer cell	KIR2DL4	0.091	0.046
Dendritic cell	BDCA-1 (CD1C)	-0.16	0.00033
Th1	STAT1	0.41	0
	IFN-γ (IFNG)	0.25	1.40E-08
Th2	GATA3	0.17	0.00027
Tfh	IL21	0.21	2.20E-06
Treg	FOXP3	0.094	0.039
	CCR8	0.14	0.0017
T cell exhaustion	PD-1 (PDCD1)	0.12	0.0086
	CTLA4	0.14	0.0028
	LAG3	0.26	1.20E-08
	TIM-3 (HAVCR2)	0.14	0.0025
	GZMB	0.15	7.00E-04

Table S7. GEPIA database verifies the correlation between ZWINT and immune cell markers.

Description	Gene markers	LUAD-ZWINT	
		Cor	P value
Neutrophils	CD66b (CEACAM8)	-0.14	0.002
	CCR7	-0.17	0.00017
Natural killer cell	KIR2DL4	0.14	0.0016
Dendritic cell	HLA-DPB1	-0.29	1.30E-10
	HLA-DQB1	-0.19	2.40E-05
	HLA-DRA	-0.26	9.10E-09
	HLA-DPA1	-0.23	6.70E-07
	BDCA-1 (CD1C)	-0.31	5.10E-12
Th1	STAT1	0.33	1.40E-13
	IFN-γ (IFNG)	0.17	0.00024
Th2	STAT6	-0.12	0.011
T cell exhaustion	LAG3	0.12	0.0078
	GZMB	0.15	0.00064
